# Therapeutic potential of natural products in ischemic stroke: targeting angiogenesis

**DOI:** 10.3389/fphar.2025.1579172

**Published:** 2025-06-18

**Authors:** WenQiang Li, E. Liu, YiHao Zhou, ZhiChao Liao, DongYan Wang

**Affiliations:** ^1^ Heilongjiang University of Chinese Medicine, Harbin, China; ^2^ Hospital of Chengdu University of Traditional Chinese Medicine, Chengdu, China; ^3^ Second Affiliated Hospital, Heilongjiang University of Chinese Medicine, Harbin, China

**Keywords:** ischemic stroke, angiogenesis, natural products, neurovascular unit, traditional medicine

## Abstract

Ischemic stroke (IS), a leading global cause of mortality and long-term disability, necessitates immediate intervention for eligible patients. Current guidelines mandate intravenous thrombolysis within 4.5 h of symptom onset for individuals without contraindications, achieving vessel recanalization rates up to 85% in treated cohorts. However, therapeutic time windows may extend to 4.5–24 h for select patients when guided by advanced multimodal imaging (CT perfusion or diffusion-weighted MRI) to identify salvageable penumbral tissue. While revascularization remains the cornerstone of acute IS management, emerging evidence underscores angiogenesis as a critical biological process for neurovascular repair and functional recovery during the subacute-to-chronic phases. Natural products (NPs), have demonstrated multiple advantages in the treatment of IS, including multi-target modulation, relatively easy access to raw materials, relatively low price, and the significant advantage of having wide selectivity offer novel opportunities for addressing these limitations. This review systematically analyses 13 major NPs classes including saponins, terpenoids, cycloenol ether terpene glycosides, flavonoids, ketones and macrocycles, phenolic acids, phenylpropanoids, polysaccharides, phenolphthaleins, complexes/extracts, volatile oils, alkaloids, and polymeric materials complexes class of compounds, We elucidate the molecular cascade in which NPs regulate the hypoxia-inducible factor 1α (HIF-1α)/Vascular endothelial growth factor (VEGF), Notch and Wnt/β-catenin signaling pathways to promote vascular regeneration. In particular, our study focuses on the critical role of angiogenesis during the development of IS, highlighting that NPs can enhance neovascularization by alleviating oxidative stress/inflammation, among other pathways. These insights offer translational possibilities for the development of NP-based combination therapies targeting both the acute neuroprotective and chronic recovery phases.

## 1 Introduction

Globally, IS is a leading cause of death and severe long-term disability. IS characterized by cerebral hypoperfusion-induced tissue necrosis (GBD, 2016 Stroke Collaborators, 2019). Clinical and epidemiological studies have identified stroke risk factors that are categorized into non-interventional and modifiable risk factors. Non-interventional risk factors include age, race, and genetic factors. Modifiable risk factors (hypertension, diabetes) contribute to 90% of stroke burden (Qi et al., 2020). According to the latest data, IS is the second leading cause of death worldwide, accounting for approximately 11 per cent of all deaths (Feigin and Owolabi, 2023). Globally, there are approximately 15 million new stroke cases each year, of which about 5 million die and another 5 million are left with permanent disabilities (Martin et al., 2025). According to the China Stroke Prevention and Treatment Report, there are approximately 3.4 million new strokes and 1.9 million deaths in China each year, making stroke the leading cause of death in China, accounting for 22.3 per cent of all deaths (Tu et al., 2023a). Strikingly, China’s hospitalization costs for stroke will be 58 billion RMB, which is a heavy burden on public finances and individuals (Tu et al., 2023b). Given China’s aging demographic trends, it is projected to be the country with the highest stroke risk in 2025 and beyond (Wardlaw et al., 2019).

Currently, clinical protocols for the treatment of IS commonly include endovascular interventions, intravenous thrombolytic therapy and neuroprotective agents, which are used to restore blood flow supply to the brain and reduce the damage caused by ischaemia. For patients with large vessel occlusion, endovascular direct intervention can be performed within a specific time window by using stent retrievers or aspiration catheters to remove the thrombus. Good collateral circulation in acute IS is beneficial for mechanical thrombectomy (Leng et al., 2016b). A good pre-treatment side branch not only improves the rate of faster and more successful recanalisation, but also increases the rate of reperfusion and is associated with lower rates of bleeding and overall mortality (Leng et al., 2016a). It is noteworthy that collateral circulation continues to demonstrate beneficial therapeutic effects even in patients who fail to achieve timely tissue-level reperfusion (Gencer et al., 2024).

Furthermore, the goal of mechanical thrombolysis is to achieve complete recanalization, which can be achieved in about 80% of patients with modern techniques (Sang et al., 2023). Second, although thrombolysis with tissue plasminogen activator (tPA) is the gold standard of IS treatment, and timely thrombolytic intervention at an early stage of treatment is effective in providing a method for cerebral thrombolysis and cerebral perfusion reconstruction (Livingston et al., 2020), tPA administration is restricted to <9.6% of patients due to hemorrhagic transformation risks (Karaszewski et al., 2009; Frank et al., 2013). The study found that semaglutide offers a potential Neurotrophic drugs therapeutic option for stroke patients. Semaglutide, a long-acting GLP-1 receptor agonist, effectively prevents the release of inflammatory components by down-regulating the expression of p65 in the NF-κB cascade. Additionally, semaglutide inhibits the expression of the M1 phenotype and promotes the expression of the M2 phenotype in microglial cells, thus reducing the neuroinflammatory cascade and promoting nerve repair, and finally exerting neuroprotective effects (Mi et al., 2024).

Drugs currently in clinical use include edaravone, whose mechanism of action is to inhibit oxidative stress and neuronal death via free radical scavengers. It is now approved by the US FDA for use in IS and has been shown in clinical trials to improve neurological function in patients with acute IS. However, it is only suitable for the acute phase (within 24–48 h of onset) and lacks evidence of long-term efficacy, and may cause renal impairment (Chen et al., 2021). Citicoline’s mechanism of action is to reduce neuroinflammation by promoting phospholipid synthesis and repairing cell membranes. Citicoline has been approved for use in stroke in Europe and Asia, and although it may improve neurological function, its efficacy is controversial (Martí-Carvajal et al., 2020). Gangliosides, such as GM1, work by enhancing neuronal plasticity and promoting axonal regeneration. They are used for acute stroke in China and some European countries, but the lack of high-quality RCT support and the possibility of inducing Guillain-Barré syndrome (rare but serious) have restricted their use in Europe and the United States (Dang et al., 2024).

The clinical translational dilemma of neuroprotective agents is a major unsolved problem in the field of stroke treatment, and the traditional view often attributes the failure to the lack of blood-brain barrier (BBB) penetration and the limitations of single-target mechanisms (Chamorro et al., 2021), but recent studies have revealed that the complexity of this problem needs to be analyzed from the dimensions of pathological heterogeneity, translational modeling defects, and experimental design deficiencies (Moustafa and Baron, 2008). Firstly, pathologic heterogeneity is complex, and the ischemic cascade involves multiple pathways, making it difficult for a single-target drug to cover the full spectrum of injury. The heterogeneity of the population is characterized by a variety of stroke etiologies with different responses to drugs, and stroke complications may alter drug metabolism and target sensitivity (Dirnagl, 2012). Secondly, The modeling flaw is that most studies in animal models use young, healthy rodents, whereas human stroke patients are mostly elderly with vascular disease, and standardized stroke models do not mimic the differences in thrombus composition and collateral circulation in human stroke (Fisher et al., 2009). Finally, trial design is flawed, with neuroprotective agents being administered 1–2 h after ischemia in animal models, whereas human trials are often delayed to 4–6 h, and there are limitations such as generalization of patient selection and implausible endpoints (Saver et al., 2013).

These challenges necessitate innovative strategies addressing both acute ischemia and chronic recovery. Recent breakthroughs identify angiogenesis as a “master regulator” of post-stroke recovery through dual mechanisms: a)Re-establishing cerebral perfusion via neovascularization (Griffioen and Dudley, 2022); b) Supporting neurogenesis through VEGF, brain-derived neurotrophic factor (BDNF) cross-talk (Xu et al., 2021). This review synthesizes contemporary evidence on NP-mediated angiogenic regulation, proposing a novel classification framework based on chemical structure-bioactivity relationships.

## 2 Stroke and angiogenesis

IS is characterized by a critical reduction in cerebral blood flow (CBF) (Zhao et al., 2022), which initiates ischemic cascades involving acidosis, glutamatergic excitotoxicity, calcium overload, neuroinflammation, and reactive oxygen species (ROS) overproduction (Yang et al., 2024). These pathophysiological events culminate in neuronal necrosis within the ischemic core and penumbra, exacerbating collateral circulation impairment and inducing irreversible neurological deficits (Li C. et al., 2021).

After IS, glutamate is released into the synaptic gap, leading to overstimulation of neuronal receptors, which leads to ischaemia and cell death, and after excitatory ischaemic injury, the overproduction of free radicals triggers oxidative and nitrosative stress, which can lead to inflammation, immune cell aggregation, and ultimately neuronal death (Zhao et al., 2024). Cerebral autoregulation is the ability of the cerebral vascular system to maintain CBF at a specific flow rate, allowing the brain to receive adequate nutrients and oxygen. In IS, cerebral autoregulation is dysfunctional and can lead to cerebral haemorrhage or cerebral ischaemia (Claassen et al., 2021). Cerebral autoregulation is a complex, nonlinear system that is regulated by many factors including, but not limited to, myogenic, neurogenic, metabolic, and endothelial mechanisms. Autoregulation maintains CBF in the brain (Armstead, 2016). Autoregulation maintains cerebral perfusion in order to sustain CBF during episodes of physiological stress. Inadequate cardiovascular reserve may exacerbate the failure of cerebral autoregulatory mechanisms if the CO or SV is impaired. The mainstay of IS management is reperfusion therapy, which is designed to save the penumbra from further tissue ischaemia and irreversible infarction (Miller et al., 2024).

Angiogenesis, defined as the sprouting of new microvasculature from pre-existing vessels, represents a pivotal compensatory mechanism under hypoxic conditions (Dudley and Griffioen, 2023). Emerging evidence identifies endothelial cells (ECs) as primary orchestrators of post-ischemic angiogenesis, coordinating with pericytes (PCs), vascular smooth muscle cells (VSMCs), and astrocytes to reconstruct microvascular networks (Yang et al., 2013). Notably, experimental studies using middle cerebral artery occlusion (MCAO) models revealed spatiotemporal upregulation of proliferating ECs and PCs in peri-infarct zones, indicative of active neovascularization (Li et al., 2010). During this process, ECs undergo cytoskeletal remodeling to form migratory tip cells guided by chemotactic cues (Jakobsson et al., 2010), while PCs and VSMCs subsequently stabilize nascent vessels through perivascular scaffolding (Quaegebeur et al., 2011). Focal cerebral angiogenesis is closely regulated by angiogenic factors, mediators and the local environment. The above factors ensure angiogenesis by correctly inducing tip cells, maintaining tip cell function and stalk cell identity, and recruiting PCs to nascent capillaries (Larrivée et al., 2009). This tightly regulated phenomenon involves dynamic interplay between angiogenic factors (e.g., VEGF, FGF), extracellular matrix (ECM) components, and astrocyte-mediated neurovascular coupling (Freitas-Andrade et al., 2020).

Angiogenesis is a key component of brain repair after stroke, and its time course is influenced by the severity of ischemia, collateral circulation, and the microenvironment, and can be divided into acute, subacute, and chronic phases (Emberson et al., 2014). In the acute phase (0–72 h) ischemic core vessels collapse and ECs in the semidiabloid zone activate, with the potential risk that the BBB is extremely permeable, and that pro-angiogenic factors may exacerbate brain edema or hemorrhage transformation, and at peak inflammation, certain proangiogenic compounds may exacerbate neuroinflammation (Albers et al., 2018). The subacute phase (3–14 days) is characterized by neovascularization and remodeling of the vascular network, and the microenvironment is characterized by subsiding inflammation, release of neurotrophic factors, and remodeling of the ECM. Whereas the optimal window for partial repair of the BBB, subsidence of inflammation, and dynamic balance between proangiogenic signaling and inhibitory signaling is reached, in the chronic phase (more than 14 days), vascular maturation (integration of functional vascular networks, glial scarring in the microenvironment, and persistent hypoperfusion in some areas) (Sun et al., 2017).

At the molecular level, the core of post-stroke angiogenesis is to restore the blood supply to the damaged brain region in order to promote the repair and functional recovery of damaged tissues. VEGF is an indispensable regulator of the angiogenic cascade and a key factor in nerve growth (Liu et al., 2014), and VEGF-A, the prototypical isoform, contributes to the regulation of angiogenesis and vascular disease (Wiszniak and Schwarz, 2021). VEGF exerts its angiogenic effects by co-ordinating multiple kinase activities through interaction with VEGF receptors (VEGFR1 and VEGFR2), which are more widely distributed in the vascular system, and VEGFR2, which is mainly found in neurons and ECs (Hu et al., 2022). Notably, although VEGF has a stronger binding affinity for VEGFR-1 than for VEGFR-2 (Wang P. et al., 2011), most of the mechanisms of VEGF are mediated through VEGFR-2. Activation of VEGFR-1 and VEGFR-2 promotes endothelial cell proliferation and migration and stimulates ECM degradation, which in turn increases vascular permeability (Facemire et al., 2009). Mechanistically, the expression of VEGF and its receptors, VEGFR-1 and VEGFR-2, was enhanced in the ischaemic hemidiaphragm region, with a significant increase in the infarcted region compared with the contralateral hemisphere (Amin et al., 2024). In addition, VEGF binding to its receptor promotes endothelial cell proliferation and migration by affecting a series of downstream signaling pathways, including the PLCγ/PKC, Ras/ERK/MAPK pathway (Koch and Claesson-Welsh, 2012). Concomitantly, basic fibroblast growth factor (bFGF) exhibits biphasic upregulation (peaking at 1 h and sustained for 14 days post-ischemia) (Huang et al., 2023), potentiating angiogenesis via direct EC stimulation and VEGF paracrine induction in VSMCs (Kanazawa et al., 2019).

Beyond the VEGF axis, a multifaceted regulatory network involving Transforming Growth Factor-beta (TGF-β), Platelet-Derived Growth Factor (PDGF), angiopoietins (Ang), and microRNAs fine-tunes angiogenic responses. TGF-β can participate in various downstream signaling pathways, both SMAD and non-SMAD. In ECs, TGF-β activates two distinct SMAD signaling pathways, Smad2/3 Smad1/5/8. The Smad2/3 pathway is triggered by ALK5 activation leading to the involvement of the Smad1/5/8 pathway, which inhibits endothelial cell proliferation, migration and angiogenesis (Hong et al., 2019). In contrast, the Smad1/5/8 pathway is triggered by activation of ALK1, which promotes endothelial cell proliferation, migration and tubule formation and angiogenesis (Zhang et al., 2021). In the non-SMAD pathway, activation of Erk1/2, TRAF4/6, PAR6 and PI3K/AKT/mTOR pathways affects vascular growth (Peng et al., 2022). PDGF are a class of serum-derived growth factors (Thomopoulos et al., 2007).

**FIGURE 1:**
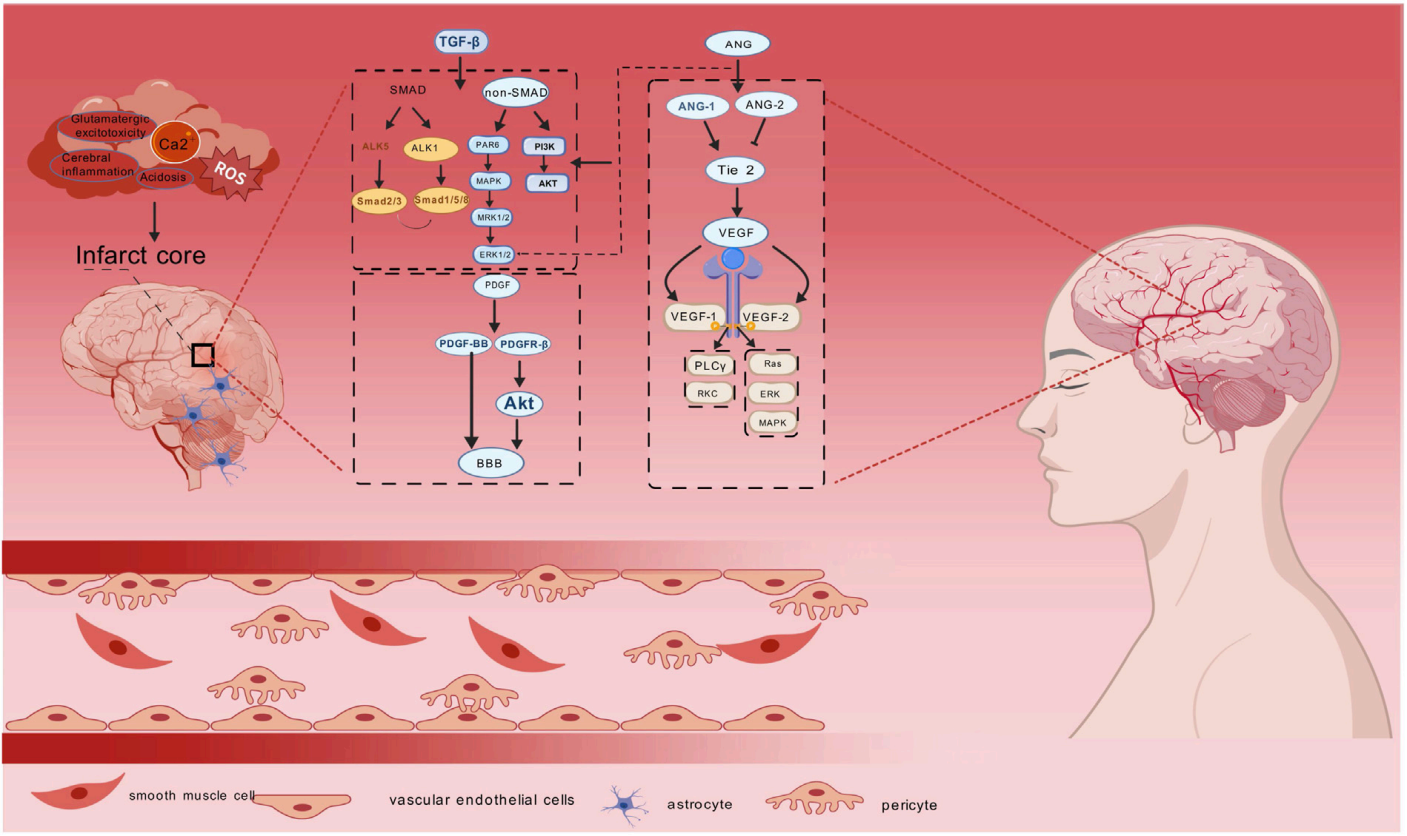
Diagram of angiogenic mechanisms in ischaemic stroke. Ischemic stroke is caused by a variety of factors, and TGF-β, PDGF, Ang, and VEGF promote tissue repair at the damaged site. TGF-β mainly affects SMAD and non-SMAD downstream signalling pathways, PDGF maintains the integrity of the BBB by regulating the PDGF-BB/PDGFR-β axis, Ang promotes cell growth and proliferation through activation of ERK1/2, PI3K/Akt and Ang-1/2, and VEGF plays apro-angiogenic role by affecting the PLCγ/PKC and Ras/ERK/MAPK pathways. Endothelial vascular cells, pericytes, smooth muscle cells and astrocytesas the main effector cells in ischaemic stroke are associated with ischaemic stroke. synergise with the above four core mechanisms to promote cerebral angiogenesis.

Notably, the PDGF-BB/PDGFR-β axis preserves BBB integrity through Akt-mediated pericyte survival (Jayaraj et al., 2019). Ang are key angiogenic mediators that play an important role in angiogenesis in brain tissue after stroke. The Ang family consists mainly of Ang-1and angiopoietin-2 (Ang-2) (Chan et al., 2016). The tyrosine kinase receptors Tie-1 and Tie-2 are associated with the expression of the ligands Ang-1 and Ang-2 (Jia et al., 2024), and Ang-2 is an inhibitor of Tie 2 signaling; once the germination pathway is established, endothelial cell proliferation and migration occurs, which is regulated through VEGF signaling, in contrast to Ang-1, which stabilises neovessels by activating the Tie 2 receptor (Wei et al., 2021). Upon receptor binding, Angtriggers multiple signaling cascades that promote cell growth, proliferation and neovascularisation through activation of ERK1/2, SAPK/JNK and PI3K/Akt-related pathways (Cucci et al., 2021). Significantly, Ang has been shown to regulate angiogenesis by stimulating endothelial cell proliferation through synergistic effects with VEGF and fibroblast growth factor (FGF), (as shown in Figure 1) (Edirisinghe et al., 2024).

In China, Chinese herbs have been used in the treatment of stroke for thousands of years, traditional Chinese medicine (TCM) has re-emerged as a promising therapeutic avenue for IS, supported by epidemiological and mechanistic studies demonstrating multi-target efficacy (Chen et al., 2012; Wang Z.-M. et al., 2023). Phytochemicals (salvianolic acids, ginsenosides) exert pleiotropic effects encompassing antioxidant, anti-inflammatory, and neurovascular protection, while enhancing angiogenesis with favorable safety profiles (Wang J. et al., 2021), and that the active ingredients of herbs and natural medicines promote angiogenesis through the above mechanisms and produce fewer side effects (Zhu et al., 2021). Astonishingly, Studies on the use of herbal monomers and NPs for the treatment of IS have focused on their active ingredients or active sites. This review systematically evaluates TCM-derived agents targeting angiogenic pathways, providing a framework for translational research bridging ethnopharmacology and cerebrovascular therapeutics.

## 3 Natural products

### 3.1 Saponin-like compound

#### 3.1.1 Ginsenosides

Ginsenosides, the principal bioactive triterpenoid saponins derived from Panax ginseng C.A. Meyer (Araliaceae family) (Yi, 2021). Exhibit a structurally diverse profile encompassing over 150 identified subtypes. Among these, ginsenoside Rg1, F1, and compound K(CK) have emerged as key mediators of neurovascular protection through multimodal mechanisms targeting inflammation suppression, oxidative stress mitigation, and vascular homeostasis regulation (Coon and Ernst, 2002). Critically, Other studies have found that ginseng and its purified ginsenoside constituents exert important effects on cardiovascular disease and its risk factors (Ye et al., 2011).

The PI3K/Akt/mTOR signaling axis, a master regulator of cellular proliferation and angiogenic programming (Zhang et al., 2016; Lee et al., 2019), is potently activated by ginsenoside Rg1. In murine stroke models, Rg1 administration significantly enhanced cerebral vascular density, evidenced by 2.3-fold increase in BrdU+/CD31+ endothelial progenitor cells (EPCs) with in peri-infarct cortices at day 14 post-treatment. *In vitro* analyses revealed Rg1-induced astrocytic vascular encapsulation through coordinated upregulation of p-Akt p-mTOR, HIF-1αand VEGF in brain microvascular endothelial cells (BMECs) (Chen J. et al., 2019).

The IGF-1/IGF1R signaling nexus, critical for neurovascular unit maintenance (Liu et al., 2001; Guan et al., 2003), is pharmacologically targeted by ginsenoside F1. Axitinib-induced vascular defects in zebrafish embryos were rescued by F1 treatment, while in rat MCAO models, F1 improved cerebral perfusion and microvascular density (MVD) IGF1R phosphorylation (Zhang et al., 2019).

Interestingly, in response to the serious challenge of low survival of mesenchymal stem cells (MSCs), CK showed a significant pro-survival effect by activating the HIF-1α/VEGF axis. Compared to MSC monotherapy, CK-MSC combination therapy in stroke models resulted in a reduction in infarct size and an increase in NeuN+/GFAP + cells (Chen et al., 2024).

Surprisingly, nanomaterial drugs have the advantage of improving the solubility, bioavailability and absorption of insoluble drugs compared to conventional drugs. Nanoporous complexes can improve the efficiency of drugs to penetrate the BBB and reach the target organ (Liu et al., 2015). To overcome the pharmacokinetic limitations of native ginsenosides, Rg1-encapsulated chitosan nanovesicles (Rg1-CNV) were engineered. *In vivo* pharmacokinetic analyses showed increased brain accumulation of Rg1-CNV compared to free drug. Mechanistically, Rg1-CNV enhanced H3K27me3 demethylation through UTX/JMJD3 recruitment, promoting endothelial migration and microvascular tube formation (Shang et al., 2022).

#### 3.1.2 Panaxatriol saponin

Panaxatriol saponin (PTS), extracted from Panax quinquefolium, primarily consists of ginsenoside Rg1, ginsenoside R1, and ginsenoside Re, which exhibit pro-angiogenic effects in HUVECs and zebrafish models (Tudor et al., 2025). In tMCAO models, PTS significantly increased VEGF and ANG-1 expression in ischemic cortical tissues, accompanied by elevated levels of endothelial cell marker CD31 and pericyte marker α-SMA. These findings suggest that PTS enhances CBF and angiogenesis, making it a promising therapeutic agent for IS(Hui et al., 2017).

#### 3.1.3 Panax ginseng leaf triterpene

Panax ginseng leaves extract (PNGL), a phytoestrogenic compoundderived from the desiccated stems and foliage of Panax ginseng C.A. Meyer has demonstrated multi-system therapeutic potential (Xie et al., 2019). Contemporary pharmacological investigations reveal its significant effects on hematopoiesis, cardiovascular homeostasis, neuromodulation, and metabolic regulation with established clinical applications in fracture healing, contusion management, and hemostasis (Xie et al., 2020).

In a rat model of MCAO/R, PNGL effectively reduced infarct volume, enhanced CBF, and induced angiogenesis (Choi et al., 2017). Mechanistic studies using human umbilical vein endothelial cells (HUMECs) revealed PNGL’s capacity to stimulate neuronal proliferation and accelerate cellular migration via dual pathway activation: the Nrf2-mediated antioxidant response and AMPK/SIRT1-dependent PGC-1α/ERα signaling axis. These findings position PNGL as a novel angiogenesis inducer with potential therapeutic applications in ischemic cerebrovascular diseases (Wang et al., 2024).

#### 3.1.4 Astragaloside IV

Astragalus membranaceus has been pharmacologically characterized to contain three principal bioactive constituents: polysaccharides, saponins, and flavonoids (Zhang J. et al., 2020). As the most representative tetracyclic triterpenoid saponin in this medicinal herb, astragaloside IV (AS-IV) exhibits diverse pharmacological properties encompassing antioxidant, anti-inflammatory, antiviral, antibacterial, antifibrotic, antidiabetic, and immunomodulatory activities (Yuan et al., 2023). Pharmacokinetic analyses in rodent models revealed dose-dependent characteristics: following intravenous administration and oral dosing, AS-IV demonstrated limited systemic bioavailability with 52.14% cumulative excretion via renal and fecal routes (Du et al., 2005; Xu et al., 2013b).

Emerging evidence implicates the PI3K/Akt/mTOR signaling axis in post-ischemic angiogenesis, with AS-IV demonstrating neurovascular protective efficacy. In MCAO models, AS-IV administration significantly attenuated cerebral histological damage, restored cortical perfusion, and enhanced MVD through pericyte-mediated vascular stabilization in peri-infarct regions. Complementary *in vitro* studies using oxygen-glucose deprived (OGD) ECs demonstrated AS-IV-induced proliferative and pro-angiogenic effects, evidenced by enhanced tube formation and PI3K inhibitor reversible VEGF upregulation, mechanistically linked to PI3K/AKT/mTOR pathway activation (G et al., 2023).

Notably, epigenetic regulation through sirtuin pathways contributes to AS-IV’s therapeutic profile. Post-infarction administration upregulated SIRT6/7 transcription, concomitant with increased CDK4/cyclin D1-mediated cell cycle progression and VEGFA/VEGFR2-driven angiogenic responses in HUVECs under OGD/reoxygenation (OGD/R) conditions. Co-immunoprecipitation assays revealed direct SIRT7-VEGFA interaction, positioning the SIRT7/VEGFA axis as a pivotal mediator of AS-IV-induced cerebrovascular remodeling (Ou et al., 2023).

miR-210 is implicated as an important target in cancer, but is also associated with cell cycle regulation, cell survival, differentiation, angiogenesis, and metabolism (Bavelloni et al., 2017). The microRNA-210 (miR-210)/HIF-VEGF-Notch regulatory network further elucidates AS-IV’s multimodal action. Cerebral ischemia upregulated miR-210 expression, which AS-IV strategically potentiated to activate HIF-1α-dependent VEGF secretion and Notch1-mediated vascular maturation in peri-infarct zones (Lou et al., 2012). In HUVEC models, AS-IV was found to activate the HIF/VEGF/Notch signaling pathway via miRNA-210. AS-IV significantly reduced the infarcted area, promoted cell proliferation and catheterization, and suppressed the expression of the target gene, hepatic alpha-collagen A3, after AS-IV intervention (Liang et al., 2020).

Peroxisome proliferator-activated receptor (PPAR) expression in ECs has been reported as early as 20 years ago, and all types of PPARs are capable of regulating angiogenesis, whereas PPARα and PPARγ have been identified to mediate anti-angiogenic processes, and the anti-angiogenic effect of PPARγ activators is mainly attributed to the increase in nitric oxide (NO) production and the opening of the sodium-potassium channel in ECs (Wagner and Wagner, 2020). PPARγ-dependent mechanisms provide additional mechanistic depth. AS-IV treatment significantly elevated M2 macrophage polarization markers and PPARγ mRNA expression, paralleled by enhanced neurogenesis and angiogenesis. Crucially, PPARγ antagonism abolished AS-IV-induced BDNF/IGF-1/VEGF upregulation, confirming PPARγ′s central role in coordinating neurovascular repair processes (Li L. et al., 2021).

### 3.2 Terpenoid compound

#### 3.2.1 Paeoniflorin

As a cornerstone herb in TCM pharmacopeia, Paeonia lactiflora Pallas (PLP) has been clinically employed for millennia in the management of nociceptive disorders, inflammatory conditions, and immune dysregulation. Pharmacological investigations have identified total glucosides of paeony (TGP), a standardized extract derived from PLP’s dried rhizomes, as its principal bioactive fraction (Zhou et al., 2020). Extensive pharmacokinetic characterization in preclinical models reveals TGP’s major active constituent, paeoniflorin (PF), exhibits limited oral bioavailability with dose-dependent tissue distribution, gastric accumulation predominates, followed by intestinal and cardiac deposition. Elimination studies have shown that more than half of the unchanged PF is eliminated by the kidneys within 24 h of IV administration, with very low faecal and biliary clearance (Liu et al., 2006).

Mechanistic studies in focal cerebral ischemia-reperfusion models elucidate PF’s multimodal neuroprotection. (1) Significant improvement in modified neurological severity scores; (2) reduction in infarct volume; (3) Restoration of regional CBF via thromboxane A2 (TXA2) suppression and prostacyclin (PGI2) preservation.

*In vitro*, PF enhanced the function of bone marrow endothelial progenitor cells (EPCs), resulting in increased proliferation, enhanced migration, and elevated tube formation compared to untreated controls. *In vivo* results showed that intraperitoneal administration of PF to rats with transient tMCAO upregulated VEGF/VEGF receptor 2 expression and increased meningeal detachment. (Liu et al., 2021).

Emerging research highlights synergistic neurovascular protection through herb-herb interaction. The canonical TCM combination of Atractylodes macrocephala Koidz (AMK) and PLP demonstrates pharmacodynamic synergism (Hu et al., 2014; Zhang X. et al., 2020). AMK-derived atractylenolides I/III (Atr I/III) potentiate PF’s effects through complementary mechanisms. (1) Atr I suppresses neuroinflammation via NF-κB inhibition; (2) Atr III enhances antioxidant capacity (More and Choi, 2017). The expression of key proteins involved in angiogenesis, IGF-2, TIMP-2, VEGFA, CXCL-4 and bFGF, which play a key role in promoting endothelial cell proliferation and migration and favoring neointimal germination, was upregulated and CCL11 and TIMP-1 were downregulated in the MACO mouse model after the intervention of Atr I, Atr III and Pae combination (Li et al., 2024). The process of angiogenesis and matrix remodeling further facilitates neuroprotection and tissue recovery in IS. ultimately promoting matrix remodeling and neurovascular repair in peri-infarct regions.

#### 3.2.2 Ginkgolide B

Ginkgo biloba extract (EGb), a globally utilized botanical drug for cardiovascular diseases, contains four principal bioactive constituents, such as ginkgolide A, ginkgolide B (GB), ginkgolide K and folium (Zhou et al., 2016). GB, a terpene lactone component of Ginkgo biloba extract, is a natural antagonist of platelet-activating factor receptors (Zhou et al., 2017). GB works by modulating microglial cell/macrophage polarisation in cerebral ischaemic injury by inhibiting anti-inflammatory effects, reducing neurological and vascular damage (Shu et al., 2016).

Emerging evidence highlights GB’s dual role in neural regeneration and vascular repair. *In vitro* and *in vivo* studies have revealed that GB potentiates the proliferation and differentiation of neural stem cells (NSCs) while reprogramming microglial polarization toward a neuroprotective phenotype (Shu et al., 2016). Mechanistically, creatine kinase B (CKB), a key regulator of vascular endothelial cell (EC) function, has been implicated in post-ischemic.

CBF dysregulation. Intriguingly, GB was found to directly bind CKB in ECs, inhibiting its enzymatic activity and subsequently activating the CCT/TRiC chaperonin complex to facilitate sphingosine kinase 1 (SK1) maturation. This SK1-dependent pathway enhances EC proliferation, migration, and tubulogenesis, ultimately promoting angiogenesis in MCAO model. (Zhu et al., 2022).

#### 3.2.3 β-asarone

β-asarone, a compound with a short plasma half-life of approximately 13 min, undergoes metabolism primarily via cytochrome P450 enzymes. Despite its broad pharmacological activities, including antithrombotic, antidepressant, anxiolytic, effects, β-asarone has been associated with hepatotoxicity, carcinogenicity, genotoxicity, and teratogenicity in toxicological studies (Chellian et al., 2017).

In MCAO model rats and OGD-treated HMEC-1 cells, β-asarone significantly increased the number of CD31^−^and Ki-67-positive cells, promoting angiogenesis through upregulation of VEGFA expression. Additionally, β-asarone upregulated endothelial nitric oxide synthase (eNOS) and induced p38 phosphorylation, further supporting its pro-angiogenic effects (Sun et al., 2024). These findings suggest that β-asarone enhances angiogenesis via the VEGFA pathway, although its clinical application remains limited due to safety concerns.

### 3.3 Cyclic enol ether terpene glycosides

#### 3.3.1 Catalpol

Catalpol, an iridoid glucoside primarily isolated from Rehmannia glutinosa (Bai et al., 2019), has emerged as a promising therapeutic candidate for cardio-cerebrovascular disorders (Zhang Z. et al., 2023). Pharmacokinetic profiling in rats reveals rapid systemic clearance, with urinary excretion as the dominant elimination pathway and tissue-specific biodistribution concentrated in the gastrointestinal tract and renal system. Notably, intact catalpol constitutes the predominant circulating species in plasma, while fecal metabolites M1/M2 suggest hepatic/intestinal metabolism (Ge et al., 2023).

Preclinical evidence underscores catalpol’s capacity to orchestrate neurovascular recovery through pleiotropic mechanisms. In MCAO models, catalpol dose-dependently reduces infarct volume and ameliorates neurological deficits by dual preservation of the neurovascular unit. (1) structural maintenance of neuron-astrocyte-endothelial tripartite interactions; (2) activation of PI3K/AKT and MEK/ERK cascades, upregulating VEGF/FAK expression to drive angiogenesis/neurogenesis (Hj et al., 2022).

Mechanistic dissection identifies that Catalpol significantly promotes stroke angiogenesis, promotes the proliferation and differentiation of NSCs in the subventricular zone, and prevents neuronal loss and astrocyte activation in the ischemic cortex or the dentate gyrus of the hippocampus *in vivo*, through a mechanism mediated by activation of the VEGF-A/KDR pathway (S et al., 2023).

At the molecular level, catalpol engages a signaling network encompassing HIF-1α/VEGF and JAK2/STAT3 pathways. In rat cerebral microvascular endothelia, HIF-1α stabilization triggers VEGF-mediated endothelial proliferation/migration, effectively restoring microcirculation in ischemic penumbras (H et al., 2020). More interestingly, catalpol’s activation of JAK2/STAT3 signaling, elevating VEGF mRNA/protein levels by 2.1 fold, normalizing CBF heterogeneity, and improving post-stroke neurobehavioral outcomes (Dong et al., 2016).

Translational relevance is evidenced in pMCAO rats receiving catalpol at 24h post-occlusion Treatment significantly enhances angiogenesis markers, such as EPO, VEGF, ameliorates neurological deficits, and mitigates cerebral capillary edema—collectively supporting its role in collateral circulation enhancement via neovascularization (Zhu et al., 2010).

#### 3.3.2 Cornus officinalis iridoid glycoside

First recorded in the Shennong Ben Cao Jing (Classic of the Materia Medica of the Divine Husbandman) about 2000 years ago, Cornus officinalis Iridoid Glycoside (CIG)is the main component extracted from Cornus officinalis Sieb. Pharmacokinetic profiling in Sprague Dawley rats revealed rapid absorption kinetics, short elimination half-life, and limited oral bioavailability (Zhang et al., 2024). In MCAO rats, the mRNA expression of VEGF and its receptor Flk-1 as well as the protein expression of VEGF were found to be significantly enhanced after CIG treatment at 7 and 28 days after ischemia. The results suggest that CIG promotes neurogenesis and angiogenesis and improves neurological function after ischemia in rats, and that the mechanism of this may be related to an increase of CIG in the brain by VEGF and Flk-1 (Yao et al., 2009).

#### 3.3.3 Cornin

Cornin, an iridoid glycoside derived from Verbena officinalis L, exhibits antioxidant properties by inhibiting lipid peroxidation (Vareed et al., 2007). In MCAO rat models, cornin administration significantly reduced cerebral infarct volume, attenuated BBB leakage, and improved neurological recovery (Lan et al., 2022). Further studies revealed that cornin promotes angiogenesis by upregulating Wnt5a, β-catenin, cyclin D1, and Ang1 expression in rat arterial smooth muscle cells (RASMC). These effects are mediated through the Ang1/Tie2 and Wnt/β-catenin pathways, thereby enhancing functional recovery post-stroke (Xu et al., 2016).

### 3.4 Flavonoids

As ubiquitous plant-derived secondary metabolites, flavonoids exhibit multimodal biological activities through ROS scavenging and redox modulation (Xu et al., 2020), its biological effects are attributed to their potential cytotoxicity and antioxidant capacity, the oxidation of flavonoids is mainly catalyzed by polyphenol oxidases (catecholoxidases and laccases) and peroxidases, with antibacterial, antioxidant and anti-inflammatory activity properties (Agati et al., 2012).

#### 3.4.1 Hydroxy saffron yellow A

Carthamus tinctorius L is a traditional Chinese herb whose preparations are widely used in cardiovascular diseases. Hydroxy saffron yellow A (HSYA)is the main active ingredient extracted from this herb. Pharmacological studies have demonstrated that HSYA can inhibit neuronal apoptosis by inhibiting the p38 MAPK signaling pathway (Li et al., 2019)and also acts as an antioxidant because of its multiple phenolic hydroxyl groups, which provide reactive hydrogen atoms to prevent oxidative damage. Experimental studies have revealed that HSYA may also improve cerebrovascular integrity by protecting ZO-1 stability. Since HIF-1α-induced NOX2 activation underlies microvascular endothelial cell destruction during cerebral infarction, HSYA rescues cerebrovascular ECs from endotoxin damage by inhibiting the HIF-1α/NOX2 signaling cascade and increasing ZO-1 expression, which is primarily due to the protection of redox homeostasis, a finding that suggests a potential clinical application of HSYA in cerebrovascular protection (Li Y. et al., 2022).

In experiments using the MCAO rat model, HSYA treatment significantly reduced infarct size, neurological scores, and cerebrovascular permeability in MCAO rats. *In vitro*, HSYA had a strong effect on cerebral vasodilation and significantly reduced platelet aggregation, blood viscosity and thrombosis, suggesting that HSYA may prevent IS by dilating cerebral blood vessels and improving cerebral vascular permeability (Sun et al., 2018). In addition, it has been shown that HSYA treatment attenuates OGD-induced cell loss by promoting proliferation and inhibiting apoptosis *in vitro*, and protects BMECs from ischemia-reperfusion injury by decreasing PHLPP-1 expression and activating Akt signaling, and by stimulating VEGF and eNOS signaling, suggesting that HSYA attenuates oxygen-glucose deprivation by down-regulating PHLPP-1. This suggests that HSYA rescues BMECs *in vitro* by down-regulating PHLPP-1 and attenuating oxygen-glucose deprivation-induced endothelial cell death in cultured brain microvessels. These multimodal actions position HSYA as a promising candidate for cerebral ischemia/reperfusion injury management (Cao et al., 2020).

#### 3.4.2 Quercetin

Quercetin is a flavonoid found in a variety of plants, such as the TCM Tiger Balm, and quercetin has strong antioxidant and anti-inflammatory activities (Cho et al., 2006). Pharmacokinetic studies reveal that quercetin demonstrates an oral clearance of 3.5 × 10^4^ L/h with a mean terminal half-life of 3.5 h (Moon et al., 2008). Emerging evidence suggests that quercetin exerts significant neuroprotective effects through multiple mechanisms. Pretreatment with quercetin has been shown to markedly upregulate endogenous antioxidant enzyme expression in hippocampal CA1 pyramidal neurons of ischemic injury models, while simultaneously preserving BBB integrity (Chen L. et al., 2019). Furthermore, quercetin demonstrates remarkable endothelial protective properties. It enhances the viability, migration, and angiogenic capacity of BMECs while suppressing apoptosis.

At the molecular level, quercetin activates the Keap1/Nrf2 signaling pathway and downregulates ATF6/GRP78 protein expression, thereby promoting cell proliferation, migration, and angiogenesis. These effects are mediated through the reduction of mitochondrial membrane potential damage and inhibition of apoptosis, which are closely associated with its antioxidant properties and suppression of endoplasmic reticulum stress (Li M.-T. et al., 2021). Notably, quercetin enhances BBB function by increasing connexin levels and potentiates mitochondrial protection through activation of mitoBKCa potassium channels, thereby inducing mitochondria-mediated cytoprotection (Kicinska et al., 2020). Collectively, these findings underscore the multifaceted protective effects of quercetin on cerebral microvascular ECs, mediated through its antioxidant, anti-apoptotic, and mitochondrial protective mechanisms.

#### 3.4.3 Baicelin

Baicalein (4,5,6-trihydroxyflavone-7-glucuronide), a naturally occurring flavonoid with potent antioxidant properties, has been demonstrated to mitigate microglial inflammatory responses (Hong and Liu, 2004). Despite its poor water solubility and low oral bioavailability due to intramolecular hydrogen bonding, baicalein exhibits favorable lipophilicity and efficient gastrointestinal absorption (Zhu et al., 2021).

Baicalin action targets reduced pro-inflammatory factors (TNF-α, IL-6, IL-1β) release, downregulated NLRP3 inflammatory vesicle activation, and inhibited caspase-1-mediated reduction of cellular focal death by inhibiting the TLR4/MyD88/NF-κB signaling axis. Experimental studies have shown that Baicalin inhibits microglia-induced neuroinflammation by suppressing NLRP3 inflammasome activation and the TLR4/NF-κB signaling pathway (Jin et al., 2019). Secondly, it reduces oxidative stress and inhibits lipid peroxidation by activating the Nrf2/ARE pathway, up-regulating antioxidant enzymes such as HO-1, superoxide dismutase (SOD), and GSH-Px, and directly scavenging ROS (Wang X. et al., 2021). It is also able to scavenge peroxynitrite (ONOO-) inhibiting nitrification stress by directly neutralizing ONOO-. Inhibits iNOS expression and reduces NO overproduction (Xu et al., 2013a). In addition, baicalein has a multi-target synergistic effect with vasoprotective and anti-apoptotic effects and promotes synaptic plasticity.

In addition to its well-established antioxidant and anti-inflammatory effects (Wang S. et al., 2011), baicalein has shown significant anti-apoptotic activity in animal models of IS(Lin et al., 2007). Pretreatment with baicalein in rat models upregulated eNOS expression while downregulating VEGF, bFGF, and inducible nitric oxide synthase (iNOS) expression. Notably, baicalein did not affect neuronal nitric oxide synthase (nNOS) expression, suggesting its neuroprotective effects are mediated through selective modulation of NOS isoforms and angiogenic molecules (Hu et al., 2005). Furthermore, studies indicate that baicalein reduces neurological deficit scores and infarct volume in MCAO models, highlighting its potential as a therapeutic candidate for ischemic cerebrovascular diseases (Qian et al., 2012).

#### 3.4.4 Apigenin

Apigenin, a widely distributed flavonoid found in vegetables and fruits such as celery, onions, tea, and grapefruit, exhibits significant neuroprotective properties. *In vitro* studies demonstrate that apigenin promotes cell proliferation and migration while inhibiting apoptosis and autophagy by modulating the expression of Caveolin-1, VEGF, Bcl-2, Caspase-3, Beclin-1, and mTOR.

*In vivo* experiments further support these findings, showing that apigenin enhances vascular endothelial cell proliferation, reduces neurobehavioral scores, and decreases cerebral infarction volume in MCAO/reperfusion rats. These effects are mediated through the upregulation of VEGFR2/CD34 double-labeled EPCs and modulation of Caveolin-1, VEGF and eNOS expression in brain tissue (Pang et al., 2018). In light of these findings, these results suggest that apigenin protects against ischemia/reperfusion injury by attenuating apoptosis and autophagy, promoting endothelial cell proliferation, and improving neurological function via the Caveolin-1/VEGF pathway.

#### 3.4.5 Caragana sinica

Caragana sinica (Chinese peashrub), a Fabaceae species widely distributed in China, Mongolia, and Tibet, contains total flavonoids in Caragana (TFC) with significant neuroprotective effects. In MCAO rat models, TFC administration attenuated neurological deficits, reduced infarct volume, and promoted angiogenesis in a dose-dependent manner. At 60 mg/kg, TFC significantly upregulated CD31, VEGF, Ang-1, HIF-1α, Dll4 and Notch1 expression, enhancing angiogenesis and improving neurological function (He et al., 2018).

### 3.5 Ketones and macrocyclic compounds

#### 3.5.1 Muscone

Muscone (3-methylcyclopentadecanone), the principal bioactive macrocyclic ketone from Moschus moschiferus (Wang J. et al., 2020), demonstrates pleiotropic neuroprotective effects including anti-neuroinflammatory antioxidant and anti-apoptotic activities (Wu et al., 2011). Mechanistically, muscone attenuated glutamate-induced apoptosis in PC12 cells and primary cortical neurons via. The animal evidence indicates that the combination of muscone and (+)-borneol can eliminate ROS accumulation and inhibit IL-1β secretion in cerebral microvascular ECs, synergistically protect claudin 5 stability and improve cerebral microvascular integrity by activating the cAMP/CREB cascade that protects the BBB function, which suggests that muscone and (+)-borneol Bcl-2/Bax ratio modulation (Zhang P. et al., 2023). Meanwhile, Muscone stimulated the release of angiogenesis-related factors from monocytes/macrophages, thus promoting angiogenesis in ECs. Can improve cerebral microvascular integrity (Li Y.-C. et al., 2022). This combinatorial regimen shows translational potential for IS management.

### 3.6 Salvianolic acids

Salvianolic acids (Sals), the most abundant and biologically active compounds derived from Salvia miltiorrhiza (Danshen), are renowned for their potent antioxidant properties (Ma et al., 2019). In a study involving male mice subjected to permanent distal dMCAO, daily administration of Sals, 5-bromo-2′-deoxyuridine, and the JAK2 inhibitor AG490 from day 1 to day 14 post-ischemia significantly enhanced neurological recovery at days 14 and 28. Sals treatment also promoted post-stroke angiogenesis, increased PCs, and improved astrocyte end-foot coverage in the peri-infarct region. Moreover, sals activated the JAK2/STAT3 signaling pathway, which was critical for its pro-angiogenic effects. Inhibition of JAK2/STAT3 signaling abolished these benefits, underscoring the pivotal role of this pathway in mediating the therapeutic effects of Sals (Li et al., 2017).

#### 3.6.1 Salvianolic acid B

Salvianolic acid B (Sal B), a water-soluble component isolated from the roots and rhizomes of Salvia miltiorrhiza Bge (family Labiatae), exhibits a broad spectrum of pharmacological activities, including antioxidant, antiplatelet aggregation, and anti-ischemic effects (Cao et al., 2008; Du et al., 2020).

Sal B has demonstrated therapeutic potential in various organs and tissues, such as the lung, heart, kidney, intestine, bone, liver, and skin, as well as in conditions like depression and spinal cord injury (He et al., 2023). One study demonstrated that Sal B significantly upregulated VEGFR2 and VEGFA expression, promoting angiogenesis both *in vivo* and *in vitro*. This effect was mediated through the induction of stanniocalcin-1 (STC1), phosphorylation of protein kinase B and mammalian target of rapamycin (mTOR), and activation of VEGFR2/VEGFA signaling in ECs, thereby improving neurovascular function after stroke (Bi et al., 2022).

### 3.7 Phenylpropanoid

#### 3.7.1 2,3,5,4′-tetrahydroxystilbene-2-O-β-d-glucoside

2,3,5,4′-Tetrahydroxystilbene-2-O-β-D-glucoside (TSG), a bioactive stilbenoid from Polygonum multiflorum rhizomes, exhibits rapid absorption (detectable in plasma within 2 min post-administration) but limited oral bioavailability due to poor intestinal absorption and rapid glucuronidation-mediated metabolism. Notably, its tissue distribution is transient, with glucuronide conjugates dominating systemic exposure, yet demonstrating an exceptional safety profile with no observed cytotoxicity in preclinical models (Wang et al., 2022).

Mechanistic studies reveal TSG’s multimodal action against cerebral ischemia-reperfusion injury. In oxygen-glucose deprivation/reoxygenation (OGD-R) models, TSG inhibited OGD-R-induced iNOS mRNA expression, which may be mediated by activation of SIRT1 and inhibition of NF-κB activation, and an *in vivo* study further demonstrated that TSG significantly reduced the volume of cerebral infarcts and the number of positive cells by TUNEL staining in the cerebral cortex (Wang et al., 2009). In an *in vitro* OGD-R model, TSG inhibited OGD-R-induced iNOS mRNA expression, which may be mediated by activation of SIRT1 and inhibition of NF-κB activation, and *in vivo* studies further demonstrated that TSG significantly reduced cerebral infarct volume and the number of positive cells by TUNEL staining in the cerebral cortex. In addition, in the MCAO model, treatment with TSG upregulated the relative expression levels of VEGF,Ang1, andAngreceptor-2 in rat brain lesions, significantly increased MVD in the brain, and upregulated CD31 expression in the ischemic penumbra (Mu et al., 2017),This pro-angiogenic reprogramming correlates with accelerated functional recovery in I/R-injured brains.

### 3.8 Polysaccharide compound

#### 3.8.1 Cistanche deserticola

Cistanche deserticola, a TCM used for over 1,000 years, exhibits neuroprotective, antioxidant, antiaging, and antifatigue effects (Ko and Leung, 2007). In MCAO rat models, total glycosides (TGs) from C. deserticola significantly increased capillary density, angiogenesis, and arteriogenesis, upregulating CD31 and α-smooth muscle actin (α-SMA) expression. (Suzuki et al., 2023). Nuclear factor E2-related factor 2 (Nrf-2)-mediated phase 2 enzymes play a crucial role in protecting neurons from oxidative stress and regulating angiogenesis. TGs enhance angiogenesis, neurogenesis, and BBB integrity in ischemic brain injury, likely through activation of the Nrf-2/Keap-1 pathway (Wang F. et al., 2020).

### 3.9 Phthalide compound

#### 3.9.1 Butylphthalide

Dl-3-n-butylphthalide (NBP), an active compound derived from Chinese celery seed. It is a small-molecule drug used in the treatment of IS in China, which is proven to ameliorate the symptoms of IS and improve the prognosis of patients. Dl-NBP is a fat-soluble substance that crosses the BBB. It is rapidly absorbed and reaches peak blood concentration in 1.25 h and has a long-lasting pharmacological effect with a half-life of 11.84 h (Chen X.-Q. et al., 2019).

Experimental evidence indicates that NBP treatment significantly stimulated angiogenesis by inducing a high production of the angiogenic growth factors VEGFA and CD31. NBP treatment not only altered the regulatory level of the hedgehog signaling pathway, but also activated the transcription factor Gli1 (Dai et al., 2023). After intranasal administration of NBP1 hour after stroke, stroke mice receiving NBP showed a significant reduction in post-stroke vascular injury and more new neurons and blood vessels in the peri-infarct area at 21 days after stroke, accompanied by improved function after focal IS in mice (M et al., 2021). Compelling experimental evidence supports that L-3-n-butylphthalide (L-NBP) significantly increased the protein and mRNA expression levels of Nrf2, HIF-1α, and VEGF in the brains of MACO/R rats, and promoted the formation of micro-vessels in the diseased areas of the brains of MACO/R rats, which had a significant beneficial effect on the cerebral I/R injury in rats, and was associated with the activation of the Nrf2/HIF-1α/VEGF signaling pathway. HIF-1α/VEGF signaling pathway, suggesting that L-NBP may be a potential therapeutic drug for brain I/R injury (Huang et al., 2021).

N-butylphthalide (NBP) has emerged as a promising treatment for acute cerebral ischemia, yet its efficacy for secondary stroke prevention is hindered by inadequate pharmacokinetic properties. NBP was structurally optimised to identify compound B4, which showed excellent neuroprotective efficacy and significant oral bioavailability. Notably, in the *in vivo* transient tMCAO model, B4 significantly reduced infarct volume, surpassing the effectiveness of NBP whereas, in the photo thrombosis (PT) model, oral B4 treatment demonstrated stronger prophylactic efficacy than NBP, which is expected to provide further justification for preclinical studies (Lu et al., 2024). After the administration of NBP intervention, experimental results confirmed that NBP treatment given within 24 h after the onset of IS rescued damaged brain tissue by enhancing angiogenesis associated with upregulation of VEGF and HIF-1α expression (Liao et al., 2009).

### 3.10 Compounds/extracts

#### 3.10.1 Renshen Shouwu extract

Renshen Shouwu (RSSW), a TCM formulation composed of Panax ginseng C.A. Mey (Renshen) and Polygonum multiflorum Thunb (fleece-flower root), is widely recognized for its neuroprotective, hepatoprotective, and hematogenic properties (Wan et al., 2015). Included in the Chinese Pharmacopoeia, RSSW is clinically used for conditions such as neurasthenia, insomnia, forgetfulness, inappetence, and chronic fatigue (Wang Y. et al., 2011). In a MCAO rat model, RSSW intervention significantly increased the number of newborn neurons while enhancing MVD in the penumbral region through angiogenesis. This effect provides nutritive blood flow and a favorable microenvironment for neurogenesis. The underlying mechanism involves that RSSW inhibits the TLR4/NF-κB/NLRP3 signaling pathway, highlighting its potential as a therapeutic target for IS(Li et al., 2020).

#### 3.10.2 Snakehead fish extract

Snakehead fish (Channa striatus), a freshwater species indigenous to many Asian countries, is traditionally valued for its medicinal properties (Sahid et al., 2018). The extract is rich in arginine, which is oxidized to citrulline, a cofactor in the conversion of eNOS to nitric oxide (NO). NO exerts protective effects by reducing platelet aggregation, enhancing vasodilation, and regulating vascular tone (Chen et al., 2005; Majewska and Gendaszewska-Darmach, 2011; Sahid et al., 2018). Administration of snakehead fish extract significantly increased VEGF and VEGFR2 expression in a dose-dependent manner, along with elevated NO levels. Mechanistic studies reveal that snakehead fish extract accelerates vascularization in IS models, highlighting its potential as a therapeutic agent (Nasution et al., 2020).

#### 3.10.3 Hairy root extract of Angelica gigas

Angelica gigas (AG), revered in traditional medicine for its therapeutic efficacy, is often referred to as the “angel plant” (Kaur et al., 2020). AG extract promotes angiogenesis and microvascular remodeling, processes regulated by VEGF, Ang-1 and Ang-2, and their receptors Tie-1 and Tie-2 (Sato et al., 1995; Patan, 2004). In MCAO rats, AG extract induced cerebral angiogenesis by upregulating VEGF, Ang1, and Tie-2 expression while enhancing tight junction proteins (ZO-1 and occludin) in the ischemic brain. These effects mitigate BBB disruption and neuronal damage, facilitating stroke recovery (Oh et al., 2015).

#### 3.10.4 Ginseng and He Shou Wu

Ginseng and He Shou Wu are herbs often used in Chinese medicine, and previous studies have shown that their extracts have neuroprotective effects on IS. In MCAO rats, treatment with these herbs increased neuronal count and cerebral MVD while downregulating proteins associated with the TLR4/NF-κB/NLRP3 inflammatory pathway, including TLR4, p-NF-κB p65, NLRP3, pro-IL-1β, IL-1β, pro-Caspase-1, and Caspase-1. These findings suggest that Ginseng and He Shou Wu enhance neurogenesis and angiogenesis by modulating inflammatory signaling (Li et al., 2020).

#### 3.10.5 Combination of Atr I, Atr III and PF

Rhizoma Atractylodis Macrocephalae and Paeoniae Alba are a classic herb pair in TCM, frequently employed in stroke treatment. In a MCAO mouse model, A strong positive correlation between IGF-2 levels and angiogenesis in ischaemic brain tissue was found in the MCAO mouse model Combination of Atr I, Atr III, and PF. *in vivo*, the combination treatment significantly promoted *In vivo*, the combination significantly promoted neurological recovery and angiogenesis and increased levels of angiogenesis-related proteins, including IGF-2; *in vitro*, the combination of Atr I, Atr III, and PF enhanced cell proliferation, promoted migration, and stimulated blood vessel formation. Accordingly, the combination of Atr I, Atr III and PF showed a significant enhancement of long-term stroke recovery in mice, possibly promoting angiogenesis by increasing the activation of the IGF-2 pathway in ischaemic brain tissue (Li et al., 2024).

#### 3.10.6 Combination of Radix Astragali and safflower

The combination of Radix Astragali and safflower (AS) is a classic TCM formulation known for its ability to promote blood circulation and resolve blood stasis. In MCAO rat models, AS treatment reduced cerebral infarction volume, alleviated neurological and histopathological damage, and inhibited apoptosis. Mechanistically, AS increased levels of PDGF-BB, erythropoietin (EPO), and TGF-β1, while decreasing serum levels of platelet factor 4, Ang-2, and tissue inhibitor of metalloproteinase-1 (TIMP-1). Additionally, AS promoted angiogenesis by upregulating VEGF and CD31 expression while downregulating prostaglandin-endoperoxide synthase 2 (PTGS2) in the hippocampus and cerebral cortex of MCAO/reperfusion (MCAO/R) rats (Xu et al., 2023).

#### 3.10.7 Salvia miltiorrhiza and Panax notoginseng

The herb pair of Salvia miltiorrhiza and Panax notoginseng is extensively used in TCM for IS treatment. *In vitro* studies demonstrated that their active components increased cell viability, reduced ROS levels, and enhanced SOD expression in oxygen-glucose deprivation/reperfusion (OGD/R)-treatedPCs.These components also reduced apoptosis, increased the Bcl-2/Bax ratio, decreased cleaved caspase-3 levels, promoted cell migration, and upregulated Ang-1, PDGFR-β, and VEGF expression. Mechanistically, Sal B, Sal D, ginsenoside R1, ginsenoside Rb1, and ginsenoside Rg1 inhibit oxidative stress and apoptosis by activating the PI3K/AKT/mTOR pathway and suppressing the JNK/ERK/p38 pathway, thereby promoting angiogenesis (Zhang et al., 2022).

### 3.11 Volatile oils and aromatic compounds

#### 3.11.1 Borneol

Borneol, a TCM with a long history of use in stroke treatment (Chen Z. et al., 2019), exhibits diverse pharmacological effects, including anti-inflammatory, analgesic, sedative, antibacterial, and antitumor properties (Ji et al., 2020), Both L-borneol and synthetic borneol demonstrate cerebroprotective effects by regulating BBB permeability. Studies have shown that borneol significantly increases serum VEGF levels, reduces tumor necrosis factor-alpha (TNF-α) levels, and improves the ultrastructure of the neuronal BBB, thereby protecting neurovascular units in MCAO rats (Dong et al., 2018).

TGF-β1 exhibits a concentration-dependent dual role in angiogenesis: low concentrations synergize with VEGF, while high concentrations inhibit VEGF-mediated effects (Dobolyi et al., 2012). In MCAO rat experiments, L-borneol reduced TGF-β1 concentration, enhanced angiogenesis, and stabilized vascular permeability by lowering matrix metalloproteinase-9 (MMP9) levels. These effects are mediated through inhibition of hypoxia-inducible factor-1α (HIF-1α), angiotensin-converting enzyme (ACE), TGF-β1, MMP9 expression, as well as modulation of the Ang1-VEGF- BDNF pathway, promoting angiogenesis-coupled neurogenesis (Ma et al., 2021).

#### 3.11.2 Benzoinum

Benzoin, a TCM used for stroke treatment, exerts its effects through targets such as phosphodiesterase 4D (PDE4D), ACE, and transthyretin (TTR), as suggested by network pharmacology and molecular docking studies. In MCAO rats, benzoin significantly upregulated VEGF, sonic hedgehog (SHH), and angiopoietin-1 (ANG-1) expression while downregulating ACE expression, promoting angiogenesis at appropriate doses. Balsamic acid, the active ingredient of benzoin, protects against ISby promoting angiogenesis via the ACE-AngI-VEGF pathway (Chen et al., 2020).

#### 3.11.3 Borneol and Ligusticum striatum DC

Borneol (BO) and Ligusticum striatum DC (LCH) are a widely used herb pair in TCM, particularly for stroke treatment due to their demonstrated efficacy. *In vitro* experiments revealed that LCH attenuated vacuolar structures, nuclear pyknosis, and neuronal loss in global cerebral ischemia-reperfusion (GCIR) mice, while BO promoted the proliferation and angiogenesis of BMECs and preserved tight junction (TJ)-related proteins after IS. The combination of BO and LCH synergistically enhanced these effects, likely through upregulation of the HIF-1α/VEGF signaling pathway. This mechanism inhibits BMEC apoptosis, maintains BBB integrity, and mitigates cerebral ischemic injury (Song et al., 2023).

### 3.12 Alkaloid

#### 3.12.1 Nigella sativa

Nigella sativa (black seed), an annual flowering plant from the Ranunculaceae family native to Southwest Asia has demonstrated therapeutic potential in various diseases, including diabetes, asthma, hypertension, chronic inflammatory disorders (Kooti et al., 2016), and gastrointestinal conditions has demonstrated therapeutic potential in various diseases, including diabetes, asthma, hypertension, chronic inflammatory disorders, and gastrointestinal conditions (Maideen et al., 2021). In experimental models, the ethanol extract of Nigella sativa reduced cerebral edema and infarct size while upregulating VEGF gene expression in a dose-dependent manner. Additionally, it increased levels of matrix metalloproteinase-9 (MMP-9), an angiogenesis-related marker, suggesting its role in improving post-ischemic recovery (Soleimannejad et al., 2017).

#### 3.12.2 Radix Aconiti Coreani and Rhizoma Typhonium

Radix Aconiti Coreani (RA) and Rhizoma Typhonium (RT), collectively known as Baifuzi, are TCMs used for stroke treatment. RA, derived from Aconitum coreanum Rapaics, is used to dispel wind and phlegm, calm epilepsy, and relieve pain. RT, derived from Typhonium giganteum Engl., exhibits similar effects along with detoxification and anti-inflammatory properties. Experimental studies have shown that RA and RT improve neurological deficits, reduce cerebral infarction incidence, and modulate inflammatory and oxidative stress responses in gerbil models. These effects are mediated through activation of the PI3K/Akt and KEAP1/Nrf2 signaling pathways, enhancing vascular endothelial function and preventing ischemic brain injury (Zhou et al., 2023).

### 3.13 Polymer materials/compounds

#### 3.13.1 Icariin and MSCs

Icariin, a core component of Epimedium, is used in TCM to treat cardiovascular diseases, osteoporosis, and sexual dysfunction (He et al., 2020). Despite its therapeutic potential, icariin exhibits low oral bioavailability due to poor water solubility, membrane permeability, and slow dissolution in biological fluids (Szabó et al., 2022). MSCs have emerged as a promising therapeutic candidate for stroke, with numerous phase I/II clinical trials confirming their safety and feasibility (Asgari Taei et al., 2022). Pharmacokinetically, icariin crosses the BBB(J et al., 2019). Mechanistic studies reveal a synergistic interaction between icariin and MSCs, with their combination promoting angiogenesis and neurogenesis while inhibiting neuronal apoptosis. These effects are mediated through the phosphorylation of PI3K and ERK1/2 pathways, leading to increased levels of VEGF, BDNF, and Bcl-2 (D et al., 2018).

#### 3.13.2 Catechol-modified modified hyaluronic acid

Conventional hydrogels have been widely used in tissues and organs. These hydrogels have a high water content, are flexible and display a tissue-like three-dimensional environment (Gu et al., 2021). However, first-generation hydrogels do not interact with tissues when applied *in vivo* and their use in focal lesions is not yet common (Cook et al., 2016). Consequently, catechol-modified modified hyaluronic acid (HAD) hydrogels is a neural stem cell-derived exosome-loaded adhesive hydrogel. It encapsulates NSC-derived Exos and maintains Exo activity.

HAD hydrogels can mimic the 3D microenvironment of tissues, induce endogenous cell adhesion and proliferation, and enhance angiogenesis in ischaemic regions (Cook et al., 2016). NSC-derived Exos encapsulated in HAD hydrogels were used for IS in a mouse model of MCAO. HAD-Exo complexes maintained Exos bioactivity and enhanced cell proliferation, angiogenesis, and anti-inflammation in ischemic regions, improving cerebral infarction and neurological function, as compared to Exo treatment. HAD hydrogels are suitable as delivery scaffolds for the sequential delivery and release of Exos in ischemic regions. HAD hydrogels are suitable as delivery scaffolds for continuous delivery and release of exons in ischaemic regions (Gu et al., 2023).

#### 3.13.3 Chitosan gel containing basic FGF

Chitosan is a bioactive material that is commonly used to mediate the delivery of neurotrophic factors to the central nervous system as it plays an active role in cell proliferation, morphogenesis and wound healing, and chitosan promotes the survival and differentiation of NSC sinto neurons (Jian et al., 2018). In the MCAO model, chitosan gel containing basic FGF was applied to the stroke cavity. The gel slowly released basic FGF, which improved the local microenvironment, activated endogenous NSCs and recruited these cells to migrate to the hemidiaphragm and the stroke cavity, and enhanced angiogenesis in the hemidiaphragm and the stroke cavity, and the results showed that bFGF-chitosan gel strongly stimulated the vascular network in and around the stroke cavity. The results showed that bFGF-chitosan gel strongly stimulated the formation of vascular networks in and around the stroke cavity, induced neurogenesis in and around the stroke cavity, and promoted partial recovery of behavioural function (Duan et al., 2024).

#### 3.13.4 SS-31-hyaluronic acid-rutin

Rutin is a natural flavonoid glycoside with pharmacological effects such as anti-inflammatory, antioxidant, and vascular resilience (Pan et al., 2019). Recent studies have shown that rutin has a strong affinity for ACE2 and can activate ACE2/Ang1-7 to exert protective effects. However, its water solubility is low and it is difficult to cross the BBB limit its clinical application (Ye et al., 2020).

It has been shown that SS-31 can easily cross the BBB and exert some neuroprotective effects due to its aromatic and cationic properties. Therefore, a polymeric micellar system with brain-targeting and ischaemic penumbra enrichment (SS-31-HAD-rutin, SHR) was constructed in a study (Cerrato et al., 2015). HAD is an ideal drug carrier with high hydrophilicity, high viscoelasticity, biodegradability, hypoallergenicity, good biocompatibility, and the ability to bind to specific receptors on the surface of cells (Sawada et al., 2020). *In vivo* and *in vitro* experiments demonstrated that SHR micelles effectively accumulate in ischemic brain regions, exerting therapeutic effects through antioxidant and anti-inflammatory mechanisms, promoting neovascularization and normalization (Zhao et al., 2023).

## 4 Conclusion and prespective

**FIGURE 2:**
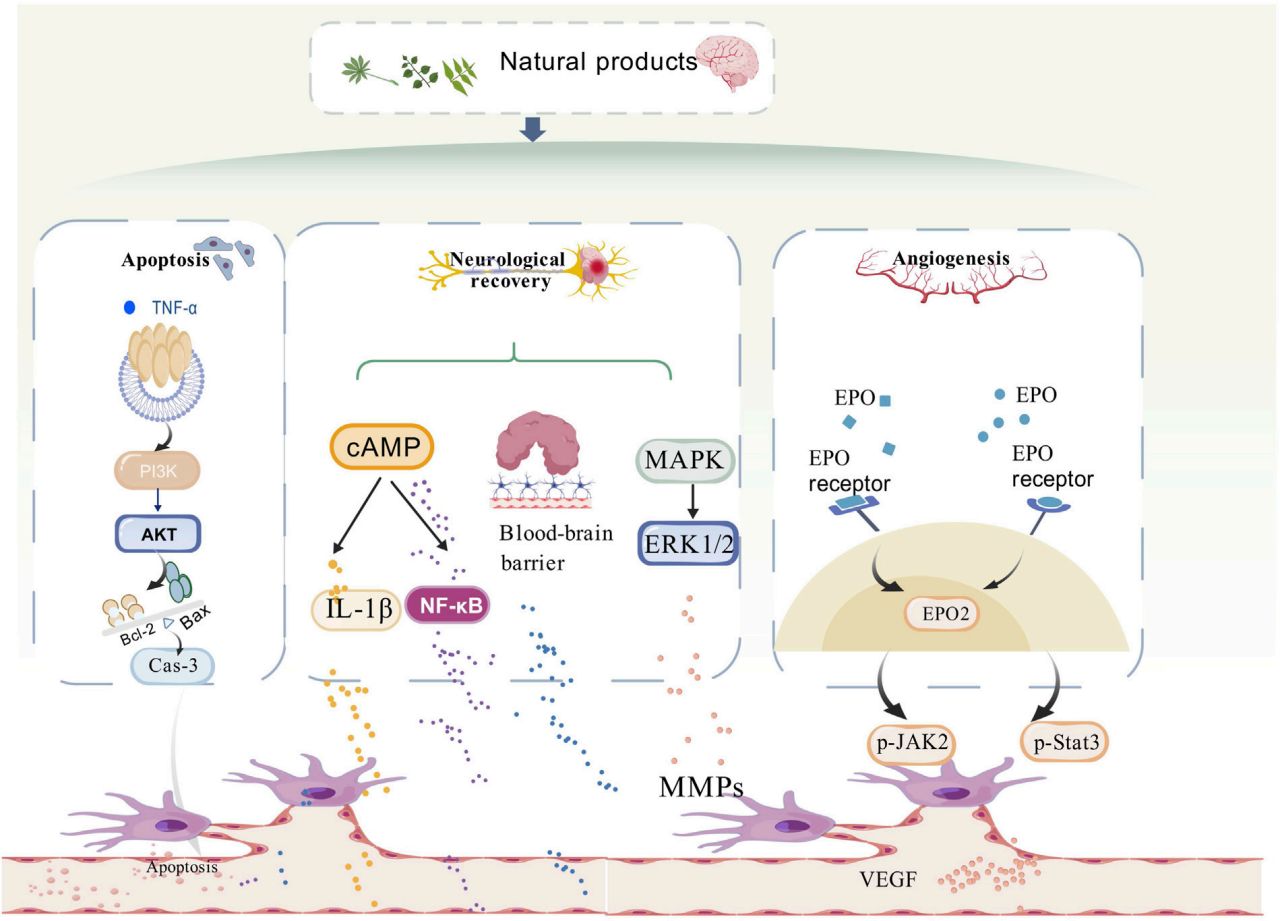
Diagram of the mechanism of natural product anti-ischemic stroke angiogenesis. Mechanistically, the mechanisms by which natural products play a role in promoting angiogenesis in stroke are related to apoptosis, neural repair, and angiogenesis. The PI3K/AKT/mTOR, ERK1/2, and JAK2/State3 signalling pathways are involved in repairing the damaged blood vessels in stroke, mainly by promoting angiogenesis.

Stroke remains one of the leading causes of disability and mortality worldwide, posing a significant threat to global health. TCM with its millennia-long clinical history in China, has demonstrated efficacy in preclinical studies, and substantial progress has been made in elucidating the mechanisms of action of herbal compounds. Currently, most of the traditional studies focus on the role of single angiogenic factors. This review highlights the role of NPs in stroke-induced angiogenesis, encompassing 13 major NP classes including saponins, terpenoids, cycloenol ether terpene glycosides, flavonoids, ketones and macrocycles, phenolic acids, phenylpropanoids, polysaccharides, phenolphthaleins, complexes/extracts, volatile oils, alkaloids, and polymeric materials complexes class of compounds. (Please refer to Figure 2). This review integrates the multi-level regulatory mechanisms of NPs on angiogenesis through multi-targeted synergistic regulatory mechanisms, including: upregulation of pro-angiogenic factors VEGF, bFGF, which through in-depth analysis of the mechanisms and cross-fertilisation of the technologies.

Although the above NPs exhibit multi-target neuroprotective potential (e.g., anti-inflammatory, antioxidant, and pro-angiogenic) in the treatment of IS, their risk of hepato-renal toxicity needs to be assessed based on the stratification of their chemical profile with respect to the dosing regimen. Low-risk components such as Ginsenosides, AS-IV and Catalpol have a favorable hepatic and renal safety profile at routine doses (Cao et al., 2023), but chronic high-dose exposure may trigger mild elevation of liver enzymes or renal tubular vacuolization through metabolic overload (Yu et al., 2025). In the intermediate-risk category, Ginkgolide B exhibited dose-dependent toxicity with 2,3,5,4′-TSG, which could exacerbate hepatocellular lipid peroxidation due to synergistic effects with anthraquinone constituents in He Shou Wu extracts (Liu et al., 2019), while Icariin may induce hepatocellular vacuolization through mitochondrial dysfunction at doses >100 mg/kg, and high-risk constituents such as β-asarone and RA need to be strictly dose-limited (Uebel et al., 2021), and their hepatotoxicity is triggered by CYP2E1-mediated active metabolites, while nephrotoxicity is associated with apoptosis of renal tubular epithelial cells.

Notably, compounded formulations may amplify the risk of toxicity due to ingredient interactions: Ginseng and He Shou Wu elevated anthraquinone bioavailability and increased the incidence of drug-induced hepatitis 2-fold (Tu et al., 2021); BO and Ligusticum synergistically induced hepatocyte apoptosis at supratherapeutic doses through activation of the BAX/BCL-2 pathway (Pang et al., 2014); furthermore, new delivery systems In addition, novel delivery systems such as chitosan gels containing basic FGF, although biocompatible, may activate hepatic Kupffer cells by long-term accumulation of their nanocarriers, potentially inducing chronic inflammation.

In terms of pharmacological models, HUVECs and male Sprague Dawley rats are the most widely utilized. Mechanistically, the VEGF/MMPs and VEGF/Akt/eNOS pathways represent key research. Unsurprisingly, derivatives of traditional medicinal plants play a pivotal role in the development of potential therapeutic formulations, with various extract forms extensively employed in proprietary Chinese medicines. (Please refer to Table 1).

**TABLE 1:** Summary of mechanisms of natural products in the treatment of ischemic stroke.

Categorization	Natural products		*In vivo*/vitro	Models	Range of dosage	Results	References
Saponin-like compound	Ginsenosides	—	*In vivo*	Male C57BL/6 mice (age 8–12 weeks, weight 22–28 g)	10,20,40 mg/kg/d	Reduction of infarct size ↑BrdU/CD31 cell Increased coverage of peripheral astrocytes	Chen et al. (2019a)
			*In vitro*	Human brain microvascular endothelial (hCMEC/D3) cell line	10 μM, 100 μM, 1,000 μM	↑p-Akt,p-mTOR,HIF-1α, VEGF	
		—	*In vivo*	Adult male Sprague Dawley rats (weighing 180–220 g)	50 mg/kg	Increased number of capillaries, vascular network formation ↑Akt,FAK, MEK1/2, eNOS、p70s6k, ERK1/2, IGF-1,IGF-1 mRNA	Zhang et al. (2019)
			*In vitro*	Human umbilical vein endothelial cells and human brain microvascular endothelial cells	20,40 μM	↑MMP-2,MMP-9, MMP-2 mRNA, MMP-9 mRNA	
		Ginsenoside CK cooperates with bone mesenchymal stem cells	*In vivo*	Sprague Dawley male rats weighing 200–220 g	28 mg/kg/day	↑ GLUT1,ATP, HIF-1α/VEG	Chen et al. (2024)
			*In vitro*	Brain microvascular endothelial-like cells	10 μM	Promote angiogenesis and vascular density and enhance neuronal and astrocyte proliferation, thereby reducing infarct size	
		Ginsenoside Rg1 complex nanovesicles (CNV)	*In vivo*	Rat brain microvascular endothelial cells	10 uM	↑JMJD3, UTX,VEGF-A,Jagged1	Shang et al. (2022)
			*In vitro*	Sprague Dawley (SD) rats	40 mg/kg/d	↑ VEGF-A,Jagged1 ↓H3K27me3	
	Panaxatriol saponin		*In vivo*	Male Sprague Dawley rats 250–300 g	50 mg/kg/day	↑VEGF, Ang-1, VEGFR-2, Tie-2, CD31, α-SMA, EC attenuates ischemic brain enhances cerebral perfusion activating the Shh pathway	Hui et al. (2017)
	Panax ginseng leaf triterpene		*In vivo*	Male Sprague Dawley	40 mg⋅kg-1	↑Nrf2, AMPK/SIRT1,PGC-1/ERα	Wang et al. (2024)
	Astragaloside IV		*In vivo*	Human brain microvascular endothelial cell line	1、2.5、10、20、50 and 100 μM	↑CD31+/BrdU +	(G et al., 2023)
			*In vitro*	Male C57BL/6 mice aged 8–10 weeks and weighing 23–25g	10,20,40 mg/kg	↑p-Akt,p-mTOR,VEGF	
			*In vivo*	Male Sprague Dawley rats, 7 weeks old	28–56 mg/kg	↑SIRT6,SIRT7,IRT6mRNA, SIRT7mRNA, VEGFA, VEGF2R, CDK4 Reduced cerebral infarction in MCAO rats	Ou et al. (2023)
			*In vitro*	HUVECs cells	16 、 32, 64 μM	↑SIRT6 、 SIRT7 、 VEGFA, VEGF2R	
			*In vivo*	Adult male Sprague Dawley (SD)rats (250–280g)w	40 mg/kg/d	↑miRNA-210 ↓ephrinA3	Liang et al. (2020)
			*In vitro*	Human umbilical vein endothelial cells	40 g/mL	↑HIF/VEGF/Notch Increased cell proliferation ability and could effec tivelyformaslendercapillary-likestructure,educed infarct size, promoted cell proliferation and ductal formation	
			*In vivo*	Male Sprague Dawley rats (10–12 weeks old, weighing 280–300 g)	40 mg/kg	↑PPARγ mRNA, BDNF,IGF-1,VEGF, improved neurological function recovery, reduced infarct volume	Li et al. (2021b)
Terpenoid compound	Paeoniflorin		*In vitro*	Endothelial progenitor cells	100 μM	Inhibit apoptosis, promote cell proliferation ratio, migration and tube formation capabilities of EPCs	Liu et al. (2021)
			*In vivo*	Rats	40 mg/kg	↑VEGF/VEGF-R	
			*In vivo*	Male C57BL/6 mice weighing 22–25g	10 μM	↑IGF-2/Ras/Raf/ERK,IGF-2/PI3K/AKT, BrdU+/CD31+	
	Ginkgolide B		*In vitro*	HUVEC	1, 3, and 9 μ M	↑CCT/TRiC-SK1 ↓CKB	Zhu et al. (2022)
	β-asarone		*In vitro*	HMEC-1	20、30, 45 μg/mL	↑ VEGFA, eNOS ↓ p-p38	Sun et al. (2024)
			*In vivo*	Adult Sprague Dawley (SD) rats,weighing 220–260 g)	30 mg/kg of β-asarone twice a day	↑CD31 ^+^, Ki-67^+^,VEGFA , eNOS ↓ p-p38	
Cyclic enol ether terpene glycosides	Catalpol		*In vivo*	Neonatal rats (1-day-old, 10-day-old), male Sprague Dawley (SD) rats180-220 g	2.5, 5.0, 10.0 mg·kg-1·day-1	↑CD31 +/EdU +,VEGF-PI3K/AKT,VEGF-MEK1/2/ERK1/2	Hj et al. (2022)
			*In vitro*	3D NVU	25、50、100 μM	↑CD31 +/EdU +,DCX+/EdU+, (p-S6), BDNF, VEGF-PI3K/AKT, VEGF-MEK1/2/ERK1/2	
			*In vivo*	*Male SD rats (weighing 220 ± 20 g)*	5 mg/kg	↑VEGF-A/KDR, p-KDR	(S et al., 2023)
			*In vitro*	BMECs	f 0.01, 0.1, 1, 10, 100, and 500 μM	↑VEGF-A/KDR	
			*In vivo*	10-day-old neonatal rats, and two-month-old male Sprague Dawley (SD) rats (180–220 g)	i.v, 2.5, 5.0, 10.0 mg kg·d−^1^	improved neurological deficits and reduced the infarct volume	(H et al., 2020)
			*In vitro*	BMECs	25,50,100 μM	↑HIF-1α/VEGF	
			*In vivo*	Healthy male Sprague Dawley (SD) rats (220–250 g)	5 mg/kg	↑EPO/EPOR/JAK2/STAT3/VEGF	Dong et al. (2016)
			*In vivo*	male Sprague Dawley (SD) rats (220∼280 g)	5 mg/kg	↑EPO 和 VEGF	Zhu et al. (2010)
	Cornus officinalis iridoid glycoside		*In vivo*	male Sprague Dawley	20,60and 180 mg/kg/day	↑VEGF, Flk-1	Yao et al. (2009)
	Cornin		*In vitro*	Rat artery smooth muscle cell	0,1, 3, 10 μM	↑Ang 1, Wnt/β-catenin ↓ cyclin D1	Xu et al. (2016)
			*In vivo*	Sprague Dawley rats	25 mg/kg	↑p-Akt,Tie2, Ang1,Wnt5a,β-catenin,cyclin D1, Ang1	
Flavonoids	Hydroxy saffron yellow A		*In vivo*	male C57BL/6J	50 mg/kg	↑IL-1β, TNF-α ↓ROS Protected Brain Microvessels against Ischemic Injury	Li et al. (2022a)
			*In vitro*	bEnd.3	10 μM	↑GSH/GSSG, TEER, NAD ^**+**^/NADH ↓HIF-1*α* Protected ZO-1 from Oxidative Damage Protected ZO-1 Protein Stability	
	Quercetin		*In vitro*	HBMECs	0.1、0.5、1、2、5、10 μmol/L	↑ Keap1/Nrf2,Claudin-5,Zonulaoccludens-1 ↓ATF6/GRP78	Li et al. (2021c)
			*In vitro*	HBMECs	1、3、10、30 μM	↑mitoBK, mitoBK_Ca_ β3	Kicinska et al. (2020)
	Baicelin		*In vivo*	Male Sprague Dawley rats, weighing 230–280 g	25, 50, 75 mg/kg	↑ eNOS ↓ VEGF 、 bFGF, iNOS	Hu et al. (2005)
	Apigenin		*In vitro*	HBMVEC	2.5、5, 10 *μ*M	↑VEGFR2/CD34, Caveolin-1, eNOS	Pang et al. (2018)
			*In vivo*	Male Sprague Dawley (SD) rats, weighing 250 ± 20 g	25 mg/kg	↑caveoloin-1,VEGF ↓Caspase-3	
	Caragana sinica		*In vivo*	Male Wistar rats (weight: 250–280 g)	15,30, 60 mg/kg	↑CD31,VEGF,Ang-1,HIF-1α,Dll4,Notch1	He et al. (2018)
Ketones and macrocyclic compounds	Muscone		*In vitro*	PC12, HUVECs	4 μM, 8 μM, 16 μM	↑THP-1, VEGF、bFGF, MMP-9, VEGFR2 decreased the number of cell lesions in the cortex and striatum and increased the number of Nissl bodies, stimulate the proliferation, migration and tube formation	Zhang et al. (2023a)
			*In vivo*	Male Sprague Dawley (SD) rats (250–280 g)	0.5,1,2,4 mg/kg	↑Bcl-2,THP-1,THP-1mRNA, VEGF ↓Bax,Bax/Bcl-2,Cleaved-caspase-3	
			*In vivo*	Male C57BL/6J mice (six to eight-week-old, 20–22 g)	2 mg/kg	↑claudin 5, CD31^+^ Protects Brain Microvessels against Ischemic Brain Injury	Li et al. (2022b)
			*In vitro*	bEnd.3	0–2 μM	↑cAMP/CREB/Claudin 5 ↓IL-1β	
Salvianolic acids	Salvianolic acids		*In vivo*	Male c57BL/6, (25–30g)	10–30 mg/kg	↑VEGF,Ang-1,Ang-2, JAK2/STAT3	Li et al. (2017)
	Salvianolic acid B		*In vitro*	HUVECs	50 μg/mL	↑CD31, STC1, AKT/mTOR, VEGF, VEGFR2	Bi et al. (2022)
			*In vivo*	Male Sprague Dawley rats (200–230 g)	10 mg/kg/d, 20 mg/kg/d	↑CD31, STC1, AKT/mTOR, VEGF, VEGFR2	
			*In vivo*	Male Sprague Dawley rats (8 weeks old; weight, 300 ± 20 g)	20 mg/kg	↑(p-)VEGFR-2,p-AKT, p-p38 MAPK VEGF, VEGFR-2	
Phenylpropanoid	2,3,5,4′-tetrahydroxystilbene-2-O-β-d-glucoside		*In vivo*	male Sprague Dawley rats	30, 60, 120 mg/kg/d	↑CD31, angiopoietin 1, angiopoietin receptor-2	Mu et al. (2017)
Polysaccharide compound	Cistanche deserticola		*In vivo*	Sprague Dawley rats 250–300g	280 mg/kg	↑CD31, α-SMA, PDGFRβ, claudin-5, occludin, ZO-1, Nrf-2 ↓Keap-1, MDA Attenuate Neuronal Injury Attenuate BBB Disruption Promote Angiogenesis Increase Expression of Tight Junction Proteins Increase Pericyte Coverage on Capillaries Alter Nrf-2 and Keap-1 Expressions	Wang et al. (2020a)
Phthalide compound	Butylphthalide		*In vivo*	Male SD rats (260–280 g) and Male C57BL/6 J mice (25–30 g)	4, 8 mg/kg, i.v	↑hedgehog	Dai et al. (2023)
			*In vitro*	,HUVECsHBMECs	0、50、100 μM	↑VEGFA,CD31	
			*In vivo*	C57BL/6	100 mg/kg	↑VEGF	(M et al., 2021)
			*In vivo*	Forty-five male Sprague Dawley rats (weighing 250–270g)	80 mg/kg, once per day	↑Nrf2,HIF-1α, VEGFmRNA	Huang et al. (2021)
			*In vivo*	260 male Sprague Dawley rats weighing 80–100 g	80 mg/kg/d	↑CD31, VEGF, HIF-1 α	Liao et al. (2009)
Compounds/extracts	Renshen Shouwu extract		*In vivo*	Healthy adult male Sprague Dawley rats weighing 250–280g	50 mg/kg,100 mg/kg	↓TLR4,pNFκB,p65/p65,NLRP3,pro-IL-1β,IL-1β,pro-Caspase-1,Caspase-1	Li et al. (2020)
			*In vitro*	bEnd.3 cells	10 μM	Promotes cell proliferation,cell migration and tube formation	Li et al. (2024)
	Snakehead fish extract		*In vivo*	Sprague Dawley strain rats	200 mg, 400 mg and 800 mg/day	↑VEGFR2, VEGF, NO	Nasution et al. (2020)
	Hairy root extract of Angelica gigas		*In vivo*	Male Sprague Dawley rats	10,25,50,100 mg/kg	↑Ang-1,Tie-2, Akt, PI3K, ZO-1,Occludin ↓VEGF	Oh et al. (2015)
	Ginseng and He Shou Wu		*In vivo*	Male C57BL/6 mice weighing 22–25g	20 mg/kg, 0.2 mL	↑BrdU^+^/CD31^+^ cell,IGF-2,TIMP-2,VEGFA,C-X-C,CXCL-4,bFGF,IGF-2/Ras/Raf/ERK, IGF-2/PI3K/AKT ↓CCL11,TIMP-1 neurological function recovery promotes angiogenesis	Li et al. (2024)
	Combination of Atractylenolide I, Atractylenolide III, and Paeoniflorin		*In vitro*	bEnd.3 cells	5,10,20 mg/kg	↓CCL11,TIMP-1,IGF-2,TIMP-2,VEGFA,CXCL-4,bFGF	Li et al. (2024)
			*In vivo*	Male C57BL/6 mice weighing 22–25g	10 μM	↑IGF-2/Ras/Raf/ERK,IGF-2/PI3K/AKT, BrdU+/CD31+	
	Radix Astragali and Safflower		*In vivo*	Adult male Sprague Dawley rats (280–300 g)	3.6 g/kg	↑PDGF-BB, EPO, TGF-β,CD31+ ↓PF4 、 Ang-2, TIMP, PTGS2 Alleviated CIRI	Xu et al. (2023)
	Salvia miltiorrhiza (SAL) and Panax notoginseng (PNS)		*In vitro*	Pericytes were subjected to OGD/R	1, 5, 10 μM	↑Ang-1,PDGFR-β,VEGF PI3K/AKT/mTOR ↓JNK/ERK/P38	Zhang et al. (2022)
Volatile oils and aromatic compounds	Borneol		*In vivo*	Male Sprague Dawley 8–10 weeks old, 250 ± 10 g	0.05、0.1、0.2 g/kg	↑VEGF,Ang-1, BDNF, CD 34, VEGFR+ ↓ ACE、MMP9、HIF1α、TGF-β1, Tie2 promote angiogenesis, against ischemic brain injury	Ma et al. (2021)
	Benzoinum		*In vivo*	Male Sprague Dawley (SD) rats	0.50 g/kg, 1.00 g/kg	↑VEGF,SHH, ANG-1 ↓ACE	Chen et al. (2020)
	Borneol and Ligusticum striatum DC		*In vivo*	Male mice weighing 18–22 g	200 mg/kg LCH and 160 mg/kg BO	↑HIF-1α/VEGF	Song et al. (2023)
			*In vitro*	BMECs	LCH groups (0.1, 0.5, 1, 5, 10, 50 μ g/mL) and BO groups (0.01, 0.05, 0.1, 0.5, 1, 5 μ g/mL)	Reduction of BMECs apoptosis, improving the integrity and function of BBB	
Alkaloid	Nigella sativa		*In vivo*	Thirty-two male Wistar rats,250 ± 20g	10–20 mg/kg	↑VEGF,HI,MMP-9	Soleimannejad et al. (2017)
	Coreani and Rhizoma Typhonii		*In vivo*	Forty healthy male Mongolian gerbils 50–70g	0.586 mg/g	↑PI3K/P-Akt, KEAP/Nrf2, VEGF, HO-1 ↓ IL-6 、 TNF-α, MDA	Zhou et al. (2023)
Polymer materials/compounds	Icariin and Mesenchymal stem cells		*In vivo*	Sprague Dawley rats 260–280 g	1 μg/mL, 5 μg/mL	↑ VEGF, BDNF, Bcl-2 improved neurological function reduced infarct volume	(D et al., 2018)
			*In vitro*	MSCs	5 μmol	↑VEGF, PI3K, ERK1/2	
			*In vivo*	Male C57BL/6 mice weighing 22–25 g	20 mg/kg, 0.2 mL	↑BrdU ^+^ /CD31+cell,IGF-2,TIMP-2,VEGFA,C-X-C,CXCL-4,bFGF, IGF-2/Ras/Raf/ERK, IGF-2/PI3K/AKT ↓CCL11, TIMP-1 neurological function recovery promotes angiogenesis	
	Catechol-modified modified hyaluronic acid		*In vivo*	Adult male mice20–25g	Exos 2 μ L, approximately 10^10^	improved the neurological function, improved cereral angiogenesis and infarct volume, improved eneurovascular unit remodelling, decreased the inflammation without tissue toxicity	Gu et al. (2023)
	Chitosan gel containing basic FGF		*In vivo*	Specific pathogen-free 250–300 g	50 mg/kg twice daily	↑Glut-1+, BrdU ^+^	Duan et al. (2024)
	SS-31-hyaluronic acid-rutin		*In vitro*	rBMEC	100 μM, yield 96%	↑CD44,Hya-1,ACE2, TFEB	Zhao et al. (2023)
			*In vivo*	Adult male Sprague Dawley rats (220–250 g	0.025 mg/kg, 0.1 mg/kg,0.5 mg/kg	↑CD31,Ki67, ACE2/Ang1-7 ↓Iba1,TNF-α	

Ang, angiopoietin; Ang-1, angiopoietin-1; Ang-2, angiopoietin-2; AG, Angelica; AS, Radix Astragali and Safflower; AS-IV, astragaloside IV; BBB, blood-brain barrier; BDNF, brain-derived growth factor; bFGF, basic fibroblast growth factor; BMECs, brain microvascular endothelial cells; BO, borneol; CKB, creatine kinase B; CIG, Cornus officinalis iridoid glycoside; ECs, endothelial vascular cells; EPCs, endothelial progenitor cells; EPO, Erythropoietin; eNOS, endothelial nitric oxide synthase; FGF, fibroblast growth factor; GB, ginkgolide B; HAD, modified hyaluronic acid; HIF-1α, hypoxia-inducible factor 1α; HUVECs, human umbilical vein endothelial cells; IGF-1, insulin-like growth factor 1; IGF1-R, insulin-like growth factor 1 receptor; IS, ischemic stroke; LCH, Ligusticum striatum DC; MCAO, middle cerebral artery occlusion; MVD, microvessel density; MSCs, mesenchymal stem cells; NPs, Natural products; NO, nitric oxide; NPs, Natural products; NSCs, neural stem cells; OGD, oxygen glucose deprivation; Oss,oligosaccharides; PCs, pericytes; PDGF, platelet-derived growth factor; PF, Paeoniflorin; PNGL, Panax ginseng leaf triterpene; PLP, Paeonia lactiflora Pallas; PPAR, Peroxisome proliferator-activated receptor; PSs, polysaccharides; PTS, Panaxatriol saponin; RASMC, arterial smooth muscle cells; ROS, reactive oxygen species; SalAB, Salvianolic acid B; Sal B, salvinorin B; TCM, Traditional Chinese Medicine; TFC, Total Flavonoids in Caragana; TGF-β, transforming growth factor-β; TGP, Total glycoside of paeony; TGs, total glycosides; TSG, 2,3,5,4′-tetrahydroxystilbene-2-O-β-d-glucoside; tPA, Tissue plasminogen activator; VEGF, Vascular endothelial growth factor; VSMCs, vascular smooth muscle cells; STC1, stannous troponin 1.

Despite the rapid advancement of chromatographic separation techniques, which have facilitated the identification of numerous NPs with pro-angiogenic effects, clinical trials validating their efficacy remain limited. NPs face multiple challenges in the clinical translation of therapeutic IS. Low bioavailability is one of the core barriers, although ginsenoside Rg3 inhibits Ca^2+^ overload and neuronal damage, its limited BBB penetration capacity makes it difficult to maintain effective therapeutic concentrations. The problem of standardized preparation is equally prominent, with large batch-to-batch variations in the active ingredients of different batches of TCMs, and strict identification standards should be established.

In addition, the complexity of the mechanism of action of NPs makes it difficult to accurately predict efficacy in preclinical studies. For example, the multi-targeting properties (e.g., antioxidant, anti-inflammatory) of NPs are significant in animal models, but metabolic differences in the human body may lead to fluctuations in effect. In the future, NPs can be combined with nano-delivery technology (e.g., CXCR4 targeting carrier) and artificial intelligence-assisted standardized production to accelerate the translation process.

By summarising and comparing the similarities and differences between neurotrophic drugs and NPs of traditional Chinese medicine, it can be found that neurotrophic drugs have the advantages of a clear mechanism of action, a clear target (e.g., edaravone against oxidative stress), more adequate clinical trial data (especially edaravone and cytarabine), and the ability to standardise the production of a dose-controllable. However, there are some drugs with significant side effects (e.g., gangliosides of Guillain-Barré risk) and most of them are only applicable to the acute phase, with limited long-term restorative effects and higher costs for patients (e.g., edaravone costs more than $10,000 for a single course of treatment).

NPs of traditional Chinese medicine have the advantages of multi-targeted effects (e.g., danshen is simultaneously anti-inflammatory, antioxidant, and improves blood flow), higher safety in long-term use (e.g., panax ginseng), lower cost, and high patient acceptance, but they also face the complexity of composition, difficulty in quality control (e.g., the active ingredient of danshen varies greatly from batch to batch), and a low level of clinical evidence (most of which are small samples or observational studies) and may interact with other drugs (e.g., ginkgo and anticoagulants). Ginkgo with anticoagulants) disadvantages. Therefore, neurotrophic drugs should be developed for multi-targeting (e.g., simultaneous inhibition of oxidative stress and inflammation), and stem cell combination therapies should be explored. For TCM NPs, modern techniques (e.g., network pharmacology) should be used to analyse the active ingredients and multi-centre RCTs should be conducted to validate the efficacy.

In the treatment of IS with NPs, although the promotion of angiogenesis is the key mechanism, potential limitations in different patient populations need to be confronted. In the future, precise regulation should be achieved to address differences in angiogenic responses, and natural epigenetic modulators (e.g., resveratrol, apigenin) can be used to reactivate silenced pro-angiogenic genes. A personalised treatment system should be further developed, integrating multi-omics data and developing angiogenic response scoring systems to predict the sensitivity of patients to treatment in advance. Adopting new imaging technology, quantitative assessment, vascular maturity, to achieve real-time adjustment of the treatment plan. In addition, spatio-temporal sequential therapy can be used, in which flavonoid NPs can be used to inhibit inflammation in the acute phase (0–3 days), saponin NPs can be used to promote angiogenesis in the sub-acute phase (3–14 days), and alkaloid NPs can be used to promote neural remodelling in the recovery phase (after 14 days). Combining the above approaches highlights the important role of NPs in IS treatment from the perspective of mechanism analysis and technological innovation.

This paper illustrates the synergistic and complementary relationship of NPs, which can achieve multi-target efficacy through sequential compounding (e.g., combining flavonoid anti-inflammatory in the acute phase with saponin pro-angiogenesis in the recovery phase), complementary efficacy, and optimisation of the delivery system (balanced delivery of lipid- and water-soluble components by nanocarriers). At present, butalbital has been approved as a neuroprotective agent, and its phase III trial has confirmed that it can improve the 90-day mRS score in patients with acute IS(Wang A. et al., 2023), while the phase II trial of salbutamol B has shown that its intravenous preparation can reduce the NIHSS score and has good hepatic and renal safety. In the future, an international multi-center phase III trial can be carried out based on butylphthalide and Sal B and its synergistic effect with endovascular therapy can be explored (Guo et al., 2022).

Meanwhile, a standardized quality control and risk monitoring system can be established to optimize the extraction conditions, to qualitatively and quantitatively analyze the active ingredients by combining with HPLC, TLC and mass spectrometry, to assess the pharmacological activity by *in vitro* and *in vivo* experiments, and to rigorously screen the raw materials and suppliers to reduce the Strict screening of raw materials and suppliers to reduce the batch size of raw materials. Strictly screen suppliers to reduce batch-to-batch variation, further promote standardization and compliance, and implement strict protocol review, subject management and data monitoring with reference to international norms (e.g., FDA, EMA) to ensure reliable results. Strengthen the technical training of testing personnel to improve their ability to identify complex ingredients. The multi-dimensional strategy (technology, management, regulation) systematically solves the quality control problems of natural product research, promotes the establishment of a precise quality control system, and provides a quantifiable and optimised pathway for clinical translation.
